# Key Mechanisms and Potential Implications of *Hericium erinaceus* in NLRP3 Inflammasome Activation by Reactive Oxygen Species during Alzheimer’s Disease

**DOI:** 10.3390/antiox10111664

**Published:** 2021-10-22

**Authors:** Marika Cordaro, Angela Trovato Salinaro, Rosalba Siracusa, Ramona D’Amico, Daniela Impellizzeri, Maria Scuto, Maria Laura Ontario, Salvatore Cuzzocrea, Rosanna Di Paola, Roberta Fusco, Vittorio Calabrese

**Affiliations:** 1 Department of Biomedical, Dental and Morphological and Functional Imaging, University of Messina, 98125 Messina, Italy; cordarom@unime.it; 2 Department of Biomedical and Biotechnological Sciences, University of Catania, 95124 Catania, Italy; trovato@unict.it (A.T.S.); mary-amir@hotmail.it (M.S.); marialaura.ontario@ontariosrl.it (M.L.O.); calabres@unict.it (V.C.); 3 Department of Chemical, Biological, Pharmaceutical, and Environmental Sciences, University of Messina, 98166 Messina, Italy; rsiracusa@unime.it (R.S.); rdamico@unime.it (R.D.); dimpellizzeri@unime.it (D.I.); salvator@unime.it (S.C.); rfusco@unime.it (R.F.)

**Keywords:** inflammasome, Alzheimer’s disease, oxidative stress

## Abstract

Alzheimer’s disease (AD) is the principal cause of dementia, and its incidence increases with age. Altered antioxidant systems and inflammation have an important role in the etiology of neurodegenerative disorders. In this study, we evaluated the effects of *Hericium erinaceus*, a nutritional mushroom with important antioxidant effects, in a rat model of AD. Animals were injected with 70 mg/Kg of AlCl3 daily for 6 weeks, and *Hericium erinaceus* was administered daily by gavage. Before the experiment’s end date, behavioral test training was performed. At the end of the study, behavioral changes were assessed, and the animals were euthanized. Brain tissues were harvested for further analysis. AlCl3 mainly accumulates in the hippocampus, the principal region of the brain involved in memory functions and learning. *Hericium erinaceus* administration reduced behavioral changes and hippocampal neuronal degeneration. Additionally, it reduced phosphorylated Tau levels, aberrant APP overexpression, and β-amyloid accumulation. Moreover, *Hericium erinaceus* decreased the pro-oxidative and pro-inflammatory hippocampal alterations induced by AD. In particular, it reduced the activation of the NLRP3 inflammasome components, usually activated by increased oxidative stress during AD. Collectively, our results showed that *Hericium erinaceus* has protective effects on behavioral alteration and histological modification associated with AD due to the modulation of the oxidative and inflammatory pathways, as well as regulating cellular brain stress.

## 1. Introduction

Alzheimer’s disease (AD) is a progressive and neurodegenerative disorder. It is the most common cause of dementia in the elderly, affecting millions of people worldwide [1,2]. Different factors are considered AD inducers, such as genetics, age, co-morbidities, and educational level [3]. In particular, the disease can be divided into two different forms: the familiar AD [4], affecting people younger than 65 years, and the sporadic AD, affecting adults older than 65 [5]. One of the principal symptoms of the disease is memory loss, which gradually worsens over the years, dividing the pathology into three stages: mild, moderate, and severe. In the beginning, memory loss is mild, but later, patients lose the ability to respond to their environment and carry on a conversation [3,6]. Brain modifications induced by AD do not directly induce death but can induce complications, including trouble swallowing and immobility. These complications, in turn, could lead to increased risks of pneumonia and malnutrition, resulting in death [7].

It has been shown that oxidative stress and inflammation are tightly linked to neurodegenerative pathologies, including AD [8,9]. The molecular pathways involve redox imbalance/oxidative stress, immune response, mitochondrial dysfunction, and neuroinflammation [10]. Increased oxidative stress significantly aggravates the hyperphosphorylated Tau protein and the deposition of β-amyloid, two signs of AD [11]. From a molecular point of view, AD is characterized by β-amyloid peptides derived from trans-amyloid precursor proteins, aggregated in amyloid plaques, and hyperphosphorylated Tau protein aggregated in neurofibrillary tangles. These plagues induce synapse loss and neuronal death [12]. Additionally, current studies have verified that redox homeostasis disruption and increased oxidative stress induce a cascade of events that include inflammasome activation [13,14]. The inflammasome is a complex of many proteins, including a receptor for damage- or pathogen-derived molecular patterns (DAMPs or PAMPs) with PYD domain-containing protein 3 (NLRP3), an adaptor with caspase activation and recruitment domain (ASC), and pro-caspase-1, which is activated by ASC [15,16]. Upon sensing DAMPs or PAMPs, the NLRP3 inflammasome recruits ASC and cleaved pro-caspase-1 in its active form, which in turn proteolytically activates pro-inflammatory cytokines [17,18,19].

Modulation of cellular stress and inflammatory pathways through small redox and anti-inflammatory molecules could be a useful approach to treat neurodegenerative diseases.

*Hericium erinaceus* mycelia and fruit bodies contain many bioactive components. For these compounds, several health-promoting properties have been described [20]. In particular, they showed antibiotic, anticarcinogenic, antidiabetic, antihypertensive, antifatigue, antihyperlipidemic, cardioprotective antisenescence, nephroprotective, and hepatoprotective effects [21]. Additionally, previous studies showed the neuroprotective action of *Hericium erinaceus* mushroom [8,9]. Moreover, it displayed antioxidant activities by increasing antioxidant enzymes activities such as superoxide dismutase (SOD), catalase (CAT), and glutathione (GSH) and also reduced malondialdehyde (MDA) levels, suggesting health-promoting effects as a result of ROS scavenging [22]. Starting from these findings, the aim of this project was to evaluate the effect of *Hericium erinaceus* administration on AD, in particular, investigating its effects on oxidative stress-induced inflammasome activation during the pathology.

## 2. Materials and Methods

### 2.1. Animals

Male Wistar rats (age: six–eight weeks, weight: 250–280 g) (Envigo, Milan, Italy) were used. Animals were housed in a controlled environment and provided with standard rodent chow (Envigo, Teklad Rodent Diet T.2018.12) and water. The University of Messina Review Board for Animal Care (OPBA) approved the study. All animal experiments agreed with the new Italian regulations (D.Lgs 2014/26), EU regulations (EU Directive 2010/63), and the ARRIVE guidelines.

### 2.2. Experimental Protocol

Aluminum (AlCl_3_) is commonly used as an AD model [2]. AlCl_3_ was intraperitoneally injected daily at the dose of 70 mg/Kg for six consecutive weeks [23].

### 2.3. Experimental Groups

Rats were randomly assigned to the following groups (n = 30 for each group):

- Control: rats were injected intraperitoneally with saline;
- Control + *Hericium erinaceus*: rats were injected intraperitoneally with saline, and *Hericium erinaceus* (200 mg/kg) was administered daily orally by gavage;
- AD group: rats were injected intraperitoneally with AlCl_3_ as already described;
- AD group + *Hericium erinaceus*: rats were injected intraperitoneally with AlCl_3_ as already described, and *Hericium erinaceus* (200 mg/kg) was administered daily orally by gavage.

*Hericium erinaceus* biomass, including mycelium and primordia, generously provided by Mycology Research Laboratories Ltd. (MRL, Luton, UK), as a commercially available product, was used for investigations.

The dose of *Hericium erinaceus* was based on previous experiments [24]. Before the experiment’s end date, behavioral test training was performed. At the end of the study, behavioral changes were assessed, and the animals were euthanized. Brain tissues were harvested for further analysis.

### 2.4. Behavioral Assessment

#### 2.4.1. Morris Water Maze (MWM)

The MWM test was employed to evaluate spatial learning and memory consolidation [25]. The percentage of distance covered and the time spent in the target quadrant were recorded.

#### 2.4.2. Elevated Plus Maze (EPM)

The EPM test was employed to evaluate memory-related processes. The behavioral test was performed as already described [26,27].

#### 2.4.3. Novel Object Recognition (NOR)

The NOR test was employed to evaluate the changes in cognitive function induced by AD. The Behavioral test was performed as already described [28]. The time spent exploring the novel object was reported according to the recognition index (RI). It was calculated by dividing the time spent investigating the novel object (TN) by the time spent exploring TN and a familiar object (TF), [RI = TN/(TN + TF)]. An RI percentage higher than 50% indicates more time spent exploring the TN; an RI percentage lower than 50% indicates more time spent exploring the TF.

### 2.5. Histological Analysis

Brain samples were collected, processed, and embedded in paraffin [29]. Sections of 7 µm in thickness were cut into longitudinal sections and stained with hematoxylin and eosin (H&E). Sections were evaluated by an experienced histopathologist using a Leica DM6 microscope (Leica Microsystems SpA, Milan, Italy) equipped with a motorized stage and associated with Leica LAS X Navigator software (Leica Microsystems SpA, Milan, Italy).

The necrosis rates of the necrotic neurons out of the total neurons were manually counted along the ipsilateral of the hippocampal CA1 region [30].

### 2.6. Western Blot Analysis

Western blots were performed on the hippocampus as previously described [31,32]. Specific primary antibodies, i.e., anti-IkB-α (Santa Cruz Biotech, sc-1643), anti-NF-kB (Santa Cruz Biotechnology, sc-8008), anti-Nrf2 (Santa Cruz Biotechnology, sc-36594), anti-NOS2 (Santa Cruz Biotechnology, sc-7271), anti-p-Tau (Santa Cruz Biotechnology, sc-32275), or anti-APP (Santa Cruz Biotechnology, sc-32277), anti-NLRP3 (Cell Signaling Technology, Danvers, MA, USA), anti-ASC (Santa Cruz Biotechnology, sc-271054), or anti-Caspase-1 (Cell Signaling Technology), were mixed in a 5% *w*/*v* nonfat dried milk solution and were incubated at 4 °C overnight. Afterward, blots were incubated with a peroxidase-conjugated bovine anti-mouse IgG secondary antibody or a peroxidase-conjugated goat anti-rabbit IgG (Jackson Immuno Research, West Grove, PA, USA) for 1 h at room temperature. Membranes were also incubated with an antibody against β-actin (Santa Cruz Biotechnology, Dallas, TX, USA) to verify that the amounts of protein were equal. Signals were detected with an enhanced chemiluminescence detection system reagent (Super-Signal West Pico Chemiluminescent Substrate, Pierce) [33,34]. The relative expression of the protein bands was quantified by densitometry with Bio-Rad ChemiDoc XRS software and standardized to β-actin levels [35]. Images of blot signals were imported to an analysis software (Image Quant TL, v2003).

### 2.7. Biochemical Analysis

Biochemical analyses were conducted on the hippocampus:

#### 2.7.1. Measurement of SOD Activity

Samples were homogenized in Tris buffer and centrifuged at 13,000 rpm. Then, TritonX-100 was added, and the solution was incubated at 4 °C and centrifuged again. Samples’ absorbance was measured for 10 min at 420 nm every 60 s [26].

#### 2.7.2. Measurement of CAT Activity

Samples were homogenized in phosphate buffer, and hydrogen peroxide was added. The absorbance was measured for 0–10 min at 240 min [26].

#### 2.7.3. Determination of GSH Levels

Samples were homogenized with phosphate buffer, and a trichloroacetic acid solution was added. The solution was centrifuged, and 5,5′-dithiobis-(2-nitrobenzoic acid) was added. GSH levels were determined using a microplate reader at 412 nm [26].

#### 2.7.4. Measurement of Nitrite Levels

Samples were homogenized in phosphate buffer, and Griess reagent was added. The solution was incubated for 30 min. The absorbance was measured at 548 nm [26].

#### 2.7.5. Measurement of Lipid Peroxidation

Thiobarbituric acid-reactant substance evaluation, a suitable indicator of lipid peroxidation, was determined on samples. The absorbance of the supernatant was detected at 532 nm [26].

#### 2.7.6. Measurement of Reactive Oxygen Species

Samples were homogenized in phosphate buffer and then incubated with 1 mM dichlorofluorescein diacetate (DCFH-DA) for 10 min at room temperature in the dark. The conversion of non-fluorescent DCFH-DA to the highly fluorescent compound 20,70-dichlorofluorescein (DCF) by esterase activity was used to monitor the presence of peroxides due to the oxidative burst in the brain [36].

### 2.8. Cytokines Measurement

Hippocampal levels of IL6, TNF-α, IL-1β, IL18, and Aβ were determined using an ELISA kit (Diaclone Research, Biosource Europe, USCN life Sciences; Abcam, Milan, Italy) [23,37,38].

### 2.9. Statistical Evaluation

All values are expressed as mean ± standard error of the mean (SEM) of N observations. For in vivo studies, N represents the number of animals used. Results were analyzed by one-way ANOVA, followed by a Bonferroni post hoc test for multiple comparisons. A *p*-value of less than 0.05 was considered significant. * *p* < 0.05 vs. control, # *p* < 0.05 vs. vehicle, ** *p* < 0.01 vs. control, ## *p* < 0.01 vs. vehicle, *** *p* < 0.001 vs. control, ### *p* < 0.001 vs. vehicle.

## 3. Results

### 3.1. Effects of Hericium erinaceus Treatment on Behavioral and Histological Alterations

**Figure 1:**
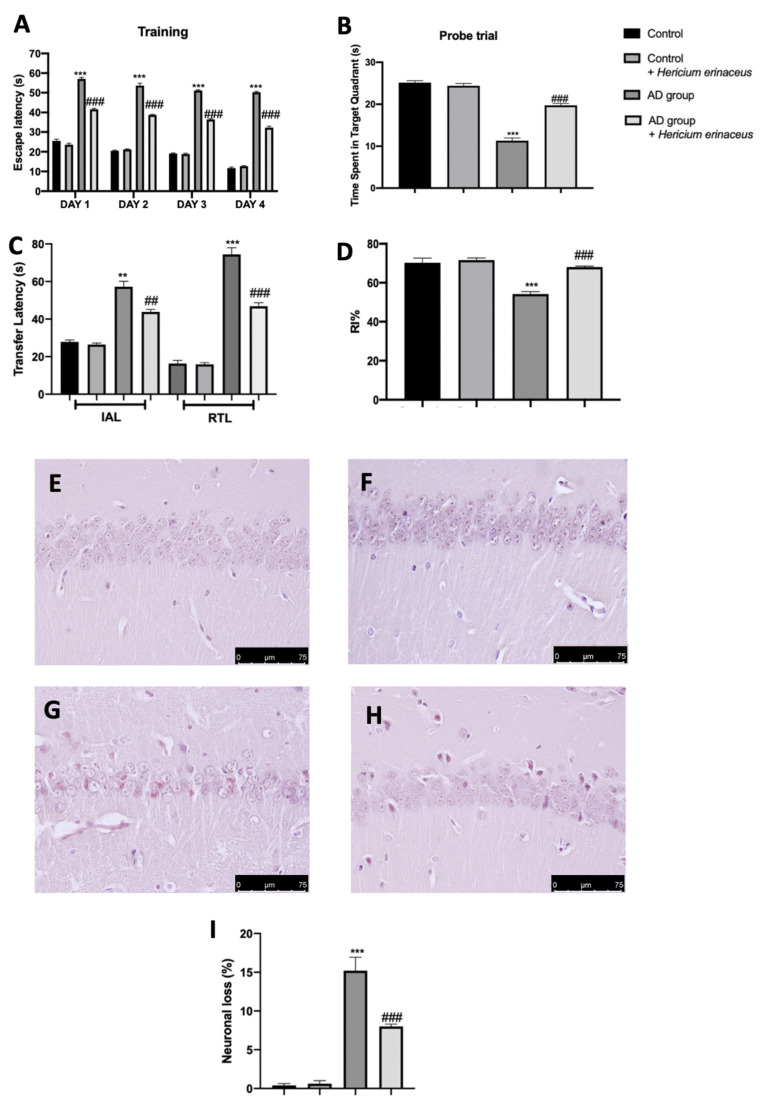
*Hericium erinaceus* administration decreased behavioral and hippocampal changes. Behavioral analyses: Morris water maze test: training (**A**), probe trial (**B**); elevated plus maze test (**C**); novel object recognition test (**D**); histological analysis: control (**E**), control + *Hericium erinaceus* (**F**), AD group (**G**), AD group + *Hericium erinaceus* (**H**); quantitative analysis of necrotic neurons (**I**). For the behavioral and histological analyses, n = 5 animals from each group and for each analysis were employed. A *p*-value of less than 0.05 was considered significant. ** *p* < 0.01 vs. control, ## *p* < 0.01 vs. vehicle, *** *p* < 0.001 vs. control, ### *p* < 0.001 vs. vehicle.

Behavioral analyses were performed to evaluate the modification induced by AD and the effects of *Hericium erinaceus* treatment. In the training period of the MWM test, on day four, the animals from all the groups displayed a decreasing trend in the escape latency time as compared to that on day one (Figure 1A). In the probe trial, *Hericium erinaceus* administration increased the animal permanence in the target quadrant, demonstrating an increase in memory consolidation as compared to the AD group (Figure 1B). In the EPM test, *Hericium erinaceus*-treated rats showed a reduced time of transfer latency in IAL and RTL, demonstrating an increase in memory retention as compared to the AD group (Figure 1C). In the NOR test, *Hericium erinaceus* treatment significantly increased the RI%, demonstrating an increase in cognitive function as compared to the AD group (Figure 1D). Brain samples collected from the control group showed normal histological structure in the CA1 hippocampal region (Figure 1E,I). In contrast, tissues harvested from the AD group showed significantly severe neuronal degeneration with evident shrunken dark basophilic neurons in the pyramidal and polymorphic layers of the CA1 hippocampal region, as well as an upregulation in glial cell infiltration (Figure 1F,I). *Hericium erinaceus* administration significantly reduced AlCl3-induced CA1 neuronal degeneration (Figure 1G,I). Additionally, both the behavioral (Figure 1A–D) and histological analysis (Figure 1F,I) did not show any difference between the control and control + *Hericium erinaceus* group; thus, we have omitted molecular analysis on the control animals administered with *Hericium erinaceus*.

### 3.2. Effects of Hericium erinaceus Treatment on APP and p-Tau Over-Expression and β-Amyloid Accumulation

**Figure 2:**
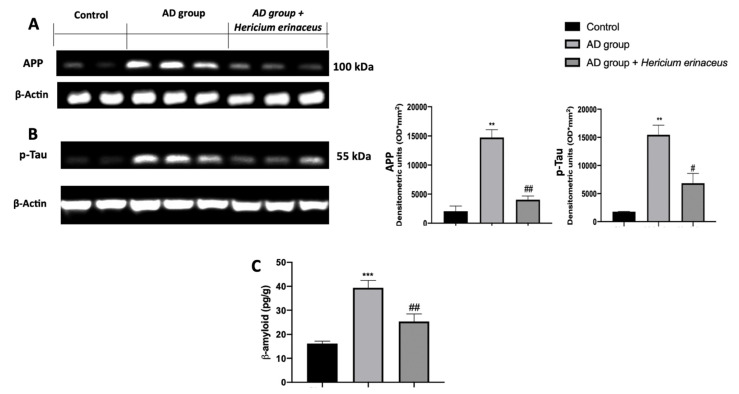
*Hericium erinaceus* administration decreased AD markers in hippocampus. Western blot analyses of amyloid precursor protein (APP) (**A**) and p-Tau (**B**) expression; β-amyloid levels (**C**). For western blot and ELISA analyses, n = 5 animals from each group and for each analysis were employed. A *p*-value of less than 0.05 was considered significant. # *p* < 0.05 vs. vehicle, ** *p* < 0.01 vs. control, ## *p* < 0.01 vs. vehicle, *** *p* < 0.001 vs. control.

Western blot analyses were conducted on hippocampal tissue to investigate the classic AD molecular markers. Increased APP (Figure 2A) and p-Tau (Figure 2B) expression was detected in tissues harvested from the AD group, as compared to the control group. *Hericium erinaceus* administration significantly decreased both levels and also reduced β-amyloid accumulation (Figure 2C).

### 3.3. Effects of Hericium erinaceus Treatment on Oxidative Hippocampal Modifications

**Figure 3:**
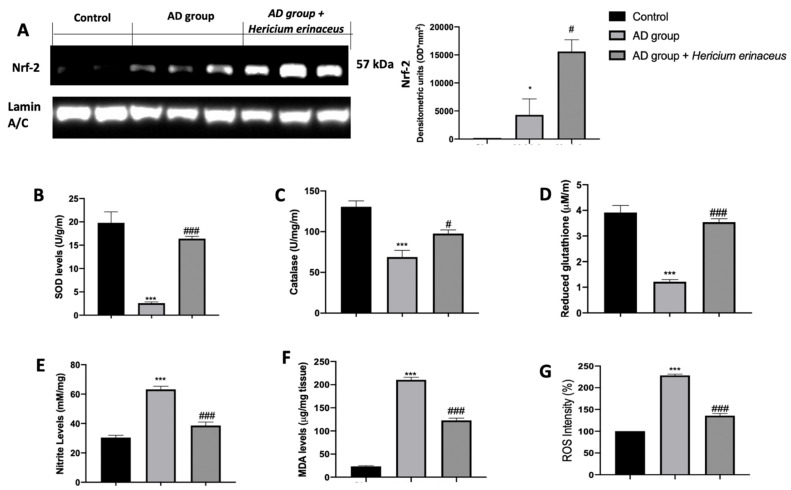
*Hericium erinaceus* administration decreased pro-oxidative modifications in hippocampus: Western blot analysis of Nrf2 expression (**A**), SOD levels (**B**), CAT activity (**C**), GSH levels (**D**), nitrite levels (**E**), MDA levels (**F**), ROS levels (**G**). For western blot and biochemical analyses, n = 5 animals from each group and for each analysis were employed. A *p*-value of less than 0.05 was considered significant. * *p* < 0.05 vs. control, # *p* < 0.05 vs. vehicle, *** *p* < 0.001 vs. control, ### *p* < 0.001 vs. vehicle.

Western blot analyses and biochemical assays were conducted on the hippocampus to investigate the antioxidant properties of *Hericium erinaceus*. Increased Nrf2 expression was detected in the hippocampus collected from *Hericium erinaceus*-treated rats, as compared to the AD group and control groups (Figure 3A). Additionally, biochemical assays showed increased antioxidant defenses in *Hericium erinaceus-*treated rats. In particular, SOD levels (Figure 3B), CAT activity (Figure 3C), and GSH levels (Figure 3D) were strongly increased in the *Hericium erinaceus* group, as compared to the AD group. Conversely, nitrite (Figure 3E), MDA (Figure 3F), and ROS (Figure 3G) levels were significantly increased in the AD group, as compared to the control group. *Hericium erinaceus* treatment considerably reduced nitrite levels, lipid peroxidation, and ROS levels in the hippocampus.

### 3.4. Effects of Hericium erinaceus Treatment on NLRP3 Inflammasome Activation

**Figure 4:**
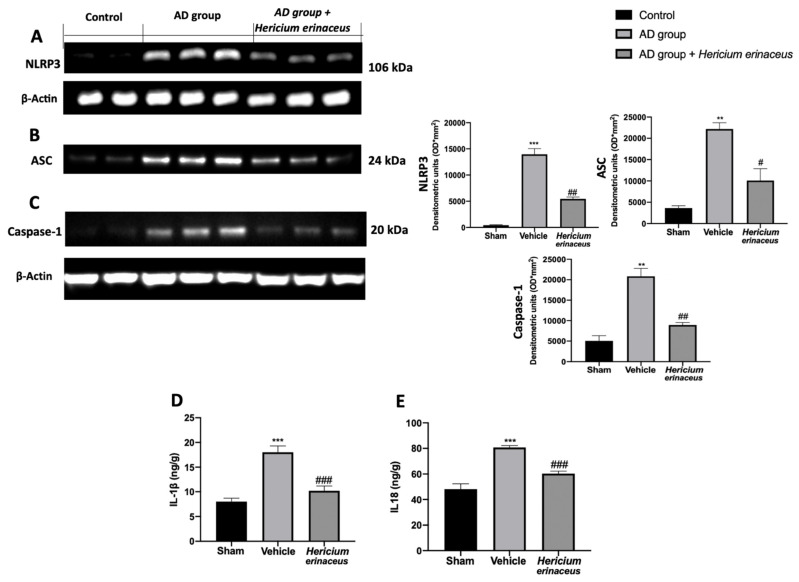
*Hericium erinaceus* administration downregulated the NLRP3 inflammasome activation in hippocampus: Western blot analyses of NLRP3 (**A**), ASC (**B**), and Caspase-1 (**C**) expression; IL1 β (**D**) and IL18 (**E**) levels. For western blot and ELISA analyses, n = 5 animals from each group and for each analysis were employed. A *p*-value of less than 0.05 was considered significant. # *p* < 0.05 vs. vehicle, ** *p* < 0.01 vs. control, ## *p* < 0.01 vs. vehicle, *** *p* < 0.001 vs. control, ### *p* < 0.001 vs. vehicle.

Western blot analyses on hippocampal tissue showed increased expression of the inflammasome components (Figure 4A–C) in the samples harvested from the AD group, as compared to the control group. *Hericium erinaceus* administration reduced NLRP3 (Figure 4A), ASC (Figure 4B), and Caspase-1 (Figure 4C) expressions. Additionally, *Hericium erinaceus* reduced IL1β (Figure 4D) and IL18 (Figure 4E) levels.

### 3.5. Effects of Hericium erinaceus Treatment on Pro-Inflammatory Parameters

**Figure 5:**
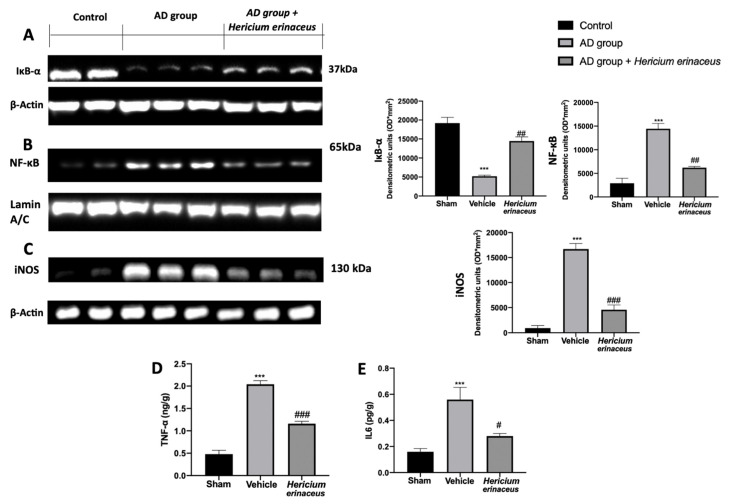
*Hericium erinaceus* administration reduced NF-kB pathway activation in hippocampus: Western blot analyses of IkB-α (**A**), NF-kB (**B**), and iNOS (**C**) expression; TNF-α (**D**) and IL6 (**E**) levels. For western blot and ELISA analyses, n = 5 animals from each group and for each analysis were employed. A *p*-value of less than 0.05 was considered significant. # *p* < 0.05 vs. vehicle, ## *p* < 0.01 vs. vehicle, *** *p* < 0.001 vs. control, ### *p* < 0.001 vs. vehicle.

*Hericium erinaceus* also showed important anti-inflammatory activities. In particular, Western blot analyses conducted on hippocampal tissue showed a strong downregulation of the NF-kB pathway, which was upregulated by AlCl_3_ injection. Samples collected from vehicle-treated rats showed reduced IkB-α expression in the cytoplasm (Figure 5A) and increased NF-kB nuclear localization (Figure 5B). *Hericium erinaceus* treatment increased IkB-α cytosolic expression and restored nuclear NF-kB expression to basal levels. Owing to the activity of the NF-kB pathway, *Hericium erinaceus* administration also reduced iNOS expression (Figure 5C) and TNF-α (Figure 5D) and IL6 (Figure 5E) levels, which were increased in the AD group.

## 4. Discussion

Despite the increasing number of studies on AD, the molecular mechanisms driving the pathology are mostly elusive, and the interventions for treating or preventing it are limited and unsuccessful.

In this paper, we evaluated the effects of *Hericium erinaceus* administration on increased reactive oxygen species and NLRP3 inflammasome activation that characterize AD.

Several papers showed the importance of oxidative stress in the progression and development of the pathology. Recent studies have shown that AD is characterized by a latent period before features emerge and diagnosis is made. In particular, the onset of AD is preceded by a mild cognitive impairment phase in which there is a small increase in β-amyloid accumulation but a significant oxidative imbalance, as compared with the healthy controls [39,40,41]. Many studies suggest that ROS overproduction induces neuronal death and related pathological changes during AD [42]. Additionally, in both in vitro and in vivo studies and in post-mortem patient brain tissues, the key role of NLRP3 inflammasome activation in β-amyloid plaque deposition and p-Tau and APP overexpression has been shown [43,44,45]. Elevated APP levels are responsible for reducing hippocampal neurogenesis and the related impaired cognitive activity [46,47]. Some evidence, in fact, showed that memory consolidation and learning and cognitive function are related to hippocampal plasticity [48,49,50].

AlCl3 neurotoxicity in animals has been clearly established and shown to be involved in the etiology of neurodegenerative diseases such as AD. It promotes the formation of amyloid-β (Aβ) protein plaques by aggregating Tau proteins in the brain. AlCl3 has also been implicated in aging-related changes and neurodegeneration. It is reported that AlCl3 toxicity is due to the increased ROS release that causes oxidative damage in the hippocampus [23]. 

AlCl3 can induce neurotoxicity via free radical production [51,52], although aluminum itself is not a transition metal and cannot catalyze redox reactions. Aluminum ions have a strong affinity for bio-membranes; it is capable of increasing the cellular oxidative milieu by potentiating the pro-oxidant properties of transition metals [53,54]. Its exposure is also associated with impairment of mitochondrial functions in vitro [55] and in vivo [56] and also impairs the antioxidant defense system, which may lead to the generation of oxidative stress [57]. Moreover, AlCl3 toxicity was found to be associated with the reduced axonal length and dendritic branches in the hippocampus [58]. Administration of AlCl3 predominantly accumulates in the hippocampus, and this region is known to be particularly susceptible to AD and has an important role in learning and memory functions [8]. For these reasons, histological, biochemical, and molecular analyses were conducted on hippocampal tissue.

*Hericium erinaceus* showed multiple positive effects on AD progression, reducing Tau over-phosphorylation, APP levels, and β-amyloid accumulation. From a behavioral point of view, it strongly reduced cognitive impairments. Histologically, it reduced the persistent hippocampal neuron loss and degeneration characteristics of AD. These behavioral and histological effects could be ascribed to the molecular properties of *Hericium erinaceus.* It activated the cellular defenses against the detrimental ROS effects by increasing the Nrf2 transcription factor and the related target genes [59]. Nrf2 is responsible for genes that encode proteins operating as endogenous antioxidant enzymes, redox balancing factors, and stress-response proteins [42]. In particular, it activated the phase II detoxifying enzymes, including Catalase, SOD, and GSH. Additionally, *Hericium erinaceus* reduced nitrite levels, lipid peroxidation, and ROS levels increased by AD [60]. These inhibitory effects on oxidative stress led to the reduction in NLRP3 inflammasome oligomerization and activity. It has already been demonstrated that antioxidants can reduce NLRP3 inflammasome activation [61]. In this paper, we have shown an important downregulation of the inflammasome complex. In particular, there is a reduced expression of the complex components NLRP3 and ASC, and Caspase-1 reduced the conversion of the pro-inflammatory interleukins pro-IL1β and pro-IL18 into their active forms. Due to this modulation of the NLRP3 inflammasome pathway, our results showed a reduction in the pro-inflammatory macroenvironment. In particular, we also showed important anti-inflammatory activities by decreasing the activation of the NF-kB pathway. NF-kB is one of the most important pro-inflammatory transcription factors [62,63,64,65,66]. Bound to its inhibitor IkB-α, in physiological conditions, NF-kB is sequestered into the cytoplasm [67,68,69]. During inflammation, the inhibitor is degraded, and NF-kB translocases into the nucleus to encode pro-inflammatory proteins [70,71]. Our results showed restored cytoplasmic levels of IkB-α and reduced NF-kB nuclear expression of the related target pro-inflammatory mediators such as TNF-α, IL6, and iNOS.

## 5. Conclusions

Thus, by decreasing oxidative stress and NLRP3 inflammasome activation, *Hericium erinaceus* manages the characteristics of AD: behavioral changes, phosphorylated Tau levels, and aberrant APP overexpression, β-amyloid accumulation, and neuronal degeneration.

## Data Availability

Data is contained within the article.
